# Sec61 in antigen cross-presentation

**DOI:** 10.18632/oncotarget.4587

**Published:** 2015-06-22

**Authors:** Matthias Zehner, Sven Burgdorf

**Affiliations:** Life and Medical Sciences (LIMES) Institute, University of Bonn, Germany

**Keywords:** Immunology and Microbiology Section, Immune response, Immunity

Upon recognition of antigens in the peripheral tissue, dendritic cells (DCs) migrate toward the draining lymph node to present internalized and processed antigens on major histocompatibility complex (MHC) molecules to T cells. In a process termed cross-presentation, antigen-derived peptides are presented on MHC I molecules to cytotoxic T cells. Cross-presentation has been shown to be of crucial importance in a variety of processes e.g. for the induction of a cytotoxic T cell response against tumors of non-hematopoietic origin or against viruses that do not infect DCs. Despite intensive investigations, the molecular mechanisms regulating cross-presentation are not fully resolved yet. In general, there are two major pathways for antigen cross-presentation: the vacuolar and the endosome-to-cytosol pathway. In the vacuolar pathway, internalized antigens are degraded and loaded onto MHC I within endosomal compartments. In the endosome-to-cytosol pathway, which is considered to be the more important one, antigens need to be transported out of the endosomes for proteasomal degradation. The molecular mechanisms facilitating such intracellular transport remained unknown for a long time and were discussed controversially [1].

In a recent study, we could demonstrate that antigen transport into the cytosol for cross-presentation is mediated by Sec61 [2]. The trimeric translocon Sec61 is a member of the ER-degredation (ERAD) machinery that enables protein transport into the ER during translation and the export of misfolded proteins from the ER into the cytosol for proteasomal degradation. We could demonstrate that upon DC activation with endotoxins, Sec61 was recruited in a TRIF-dependent manner toward antigen-containing endosomes. Down-regulation of both essential subunits Sec61α1 and Sec61γ resulted in impaired antigen export into the cytosol and hence in reduced cross-presentation [2], pointing out that Sec61 might indeed be involved in the regulation of such intracellular antigen transport.

Of crucial importance to demonstrate a decisive role of Sec61 in antigen translocation was an approach to trap Sec61 in the ER and to prevent its recruitment toward antigen-containing endosomes by the generation of a Sec61-specific ER-retained antibody (intrabody). To this end, we isolated a single chain variable fragment (scFv) antibody against the first luminal loop of Sec61α and expressed it provided with an ER targeting signal peptide and a KDEL ER retention signal in DCs. Due to its specific binding to Sec61 and its ER retention signal, it prevented Sec61 recruitment towards antigen-containing endosomes without affecting its functionality in the ER or without influencing the transport of other ER proteins toward endosomes. Such specific exclusion of Sec61 from endosomes resulted in impaired antigen translocation into the cytosol and hence cross-presentation, which was shown for a variety of antigens (including soluble and particular ovalbumin, Δ-lactamase, cytochrome C and BSA) and which unequivocally proved that endosomal Sec61 mediates antigen translocation into the cytosol for cross-presentation.

In a recent study, it was reported that cross-presentation of synthetic long peptides by human DCs was independent of Sec61, since down-regulation of Sec61α1 resulted in a moderate but non-significant reduction in cross-presentation [3]. Efficient down-regulation was analyzed by western blot, showing a reduced expression of a protein about 95 kDa. However, since Sec61α migrates slightly above 40 kDa, and the protein depicted on the western blot was also reduced after downregulation of p97 and Derlin-1, the effect on cross-presentation in this study remains unclear. Nevertheless, it is not unreasonable to think that Sec61 might not be the only translocon enabling antigen translocation into the cytosol, a notion that might be further supported by observations demonstrating that dislocation at the ER can also be mediated by Hrd1, independent of Sec61 [4]. Although Hrd1 does not seem to play a specific role in cross-presentation [2][5], it is thinkable that in addition to Sec61, other channel proteins might mediate antigen translocation from endosomes into the cytosol [5]. Future experiments will have to reveal whether such alternative translocons exist and might enhance our understanding of the exact recruitment of Sec61 from the ER toward antigen-containing endosomes.
